# Comparison of Color Stability Between PMMA and Graphene-Reinforced PMMA After Being Subjected to pH Changes and Coffee

**DOI:** 10.3390/dj14060319

**Published:** 2026-05-22

**Authors:** Ildefonso Serrano-Belmonte, Sergi Torné-Durán, Javier San Nicolás-Sánchez, Virginia Pérez-Fernández, Ascensión Martínez-Cánovas

**Affiliations:** 1 School of Dentistry, University Dental Clinic, Hospital Morales Meseguer, Faculty of Medicine, University of Murcia, 30100 Murcia, Spain; iserrano@um.es (I.S.-B.); jsannicolas2000@gmail.com (J.S.N.-S.); ascension.martinez4@um.es (A.M.-C.); 2 School of Dentistry, Faculty of Medicine and Health Science, Universitat de Barcelona, 08907 L’Hospitalet de Llobregat, Spain; 3 Departament of Sociosanitary Sciences, Institute of Biomedical Research (IMIB-Arrixaca), University of Murcia, 30120 Murcia, Spain; virperez@um.es

**Keywords:** color stability, coffee, graphene, pH, polymethyl methacrylate

## Abstract

**Background/Objectives**: Provisional restorations are widely used in fixed prosthodontics, and polymethyl methacrylate (PMMA) remains one of the most commonly used materials. Discoloration during intraoral service may compromise their esthetic acceptability. Graphene-reinforced PMMA has been introduced to improve material performance, although its color stability under simulated oral conditions remains insufficiently characterized. This study aimed to compare the color changes in conventional PMMA and graphene-reinforced PMMA after exposure to salivary pH variations and coffee, combined with simulated masticatory movements. **Methods**: Forty PMMA and forty graphene-reinforced PMMA (G-CAM) disk-shaped specimens (8 mm × 2 mm) were allocated to eight original experimental groups according to material and condition (pH 7, coffee, pH change, or pH change plus coffee), under simulated chewing movements. Color was recorded before and after the procedures using an imaging system, and values were converted into CIELAB coordinates. Statistical analysis included Wilcoxon, Mann–Whitney U, Kruskal–Wallis, and Bonferroni-adjusted pairwise comparisons, with a significance level set at *p* < 0.05. **Results**: Both materials exhibited measurable color changes after the experimental procedures. PMMA showed higher median ΔE values than graphene-reinforced PMMA in the coffee and pH change plus coffee conditions, where the differences between materials were statistically significant. The highest color changes were observed in PMMA exposed to coffee and to the combined pH change plus coffee protocol, exceeding the clinical acceptability threshold. **Conclusions**: Graphene-reinforced PMMA (G-CAM) showed greater color stability than conventional PMMA, although neither material can be considered completely color stable. The greatest color variation was observed in the groups exposed to pH change followed by coffee immersion, as reflected by ΔE values.

## 1. Introduction

Esthetics has long been a primary concern in dentistry, both in definitive and provisional restorative materials. On some occasions, provisional materials must be kept for very long periods of time, maintaining stable physical and esthetic properties [1,2]. Graphene has been investigated as a reinforcing additive for PMMA due to its high mechanical strength and large surface area, which may enhance the mechanical and surface performance of polymer-based dental materials when properly dispersed within the matrix [3,4]. In graphene-reinforced PMMA, these characteristics are expected to improve mechanical behavior and surface stability. However, graphene’s intrinsic optical properties may also influence color behavior, making color stability a relevant parameter for investigation.

One of the treatments in which provisional materials are most frequently used is fixed prosthodontics. Among these materials, polymethyl methacrylate (PMMA) has been widely employed due to its ease of handling, low cost, and acceptable mechanical properties. However, PMMA also presents several limitations, including suboptimal surface characteristics that promote bacterial adhesion, relatively low resistance to wear and fracture under masticatory forces, and susceptibility to discoloration over time. These limitations restrict its long-term clinical performance and have motivated the development of modified PMMA-based materials with improved properties [5].

One of these combinations gives rise to graphene-reinforced PMMA (G-CAM), a graphene-reinforced PMMA material specifically developed for CAD-CAM (computer-aided design and computer-aided manufacturing) provisional restorations. The incorporation of graphene is expected to act as a structural reinforcement within the polymer matrix, improving fracture resistance and fatigue behavior and contributing to a more stable mechanical response under masticatory loading. In addition, graphene-based fillers may influence surface behavior and chemical stability by reducing water sorption and limiting the adhesion of microorganisms, which are factors closely related to discoloration and surface degradation over time [6]. These potential advantages make graphene-reinforced PMMA an attractive option for long-term provisional restorations in situations where both mechanical performance and esthetic stability are critical. It is important to note that the incorporation of graphene into PMMA does not compromise the initial esthetic appearance of this material. However, the influence of graphene incorporation on the optical properties and long-term color stability of PMMA-based materials remains insufficiently understood.

The materials located in the oral cavity are subjected to numerous interactions during daily intraoral function, including food intake, the application of chewing forces and constant contact with saliva, factors that could cause changes in the surface of these materials. One issue to consider is the constant variations in pH. Although naturally everyone has a different value (it may be more acidic or more basic), it is during feeding that the salivary pH is caused to decrease to pH < 5.5, followed by the buffering of the same, reaching between pH 6.5 and pH 7.4 [6].

Simulated chewing movements may influence color stability by increasing surface wear and roughness, facilitating the penetration and retention of staining substances on the polymer surface. Therefore, incorporating masticatory simulation helps reproduce more realistic intraoral conditions that can contribute to discoloration.

Masticatory activity varies among individuals for food preparation, but the rhythm and number of chewing cycles can vary, depending on the hardness and consistency of the food. These movements can range from 17 to 63 cycles per minute [7], although in very fast movements, we can reach up to 120 cycles per minute [8].

Among the various staining agents present in daily dietary habits, coffee is one of the most widely consumed beverages worldwide and is known for its high staining potential. This effect is mainly attributed to its content of chromogenic agents, such as tannins and polyphenols, which can penetrate and adhere to polymer surfaces, leading to discoloration over time. Therefore, coffee was selected as a clinically relevant staining medium to evaluate the color stability of the materials under study.

Several investigations have reported that staining beverages such as coffee produce significant discoloration in acrylic and resin-based provisional materials due to their high concentration of tannins and polyphenolic pigments, which readily penetrate and adhere to polymer surfaces [9,10]. In addition, changes in salivary pH may contribute to surface softening or increased water sorption of PMMA-based materials, further facilitating pigment uptake over time [11]. These findings support the relevance of evaluating the behavior of both PMMA and graphene-reinforced PMMA when subjected to pH variations and coffee exposure under simulated oral conditions.

Surface characteristics, particularly roughness and wettability, play a critical role in discoloration phenomena. However, the surface roughness, topography, and wettability of the same CAD-CAM PMMA and graphene-reinforced PMMA specimens used in the present study have been previously characterized under identical manufacturing conditions, demonstrating significantly lower roughness and higher wettability for graphene-reinforced PMMA [11].

Because the present investigation uses these same previously characterized specimens, the analysis focuses specifically on the comparative color behavior of the materials under simulated oral conditions, rather than on intrinsic surface characterization.

This study aimed to compare the color changes in conventional PMMA and graphene-reinforced PMMA after exposure to salivary pH variations and coffee, combined with simulated masticatory movements, using CIELAB color analysis and ΔE evaluation. Color stability in dental materials is commonly assessed using the CIELAB color system, which allows quantitative evaluation of color differences through the ΔE parameter. This approach provides clinically relevant information by enabling interpretation of color changes in terms of perceptibility and acceptability thresholds. Therefore, incorporating CIELAB-based analysis is essential to accurately assess the esthetic performance of provisional materials.

The working hypothesis of this study was that graphene-reinforced PMMA would exhibit lower color change (ΔE) than conventional PMMA under different experimental conditions. The null hypothesis was that no significant differences in color change would be observed between the materials.

## 2. Materials and Methods

### 2.1. Sample Preparation

A total of 80 samples (40 of each material) with a cylindrical shape and 8 mm in diameter and 2 mm in height were used for the in vitro study. The PMMA and graphene-reinforced PMMA specimens analyzed in the present study were manufactured by Graphenano Dental S.L. (Valencia, Spain) from the same production batch and using identical CAD-CAM machining parameters [11]. The graphene-reinforced PMMA material (G-CAM) contained 1% < wt% of graphene, as specified by the manufacturer.

The samples were divided into 8 groups of 10 samples according to the material and the procedure to be performed (Table 1). For clarity, the numerical group codes correspond to specific combinations of material (PMMA or graphene-reinforced PMMA (G-CAM)) and experimental condition (pH variation, coffee immersion, or both), as detailed in Table 1. This nomenclature was selected to simplify the comparison of procedures throughout the study. Each specimen was assigned to a single experimental group and was not reused in additional procedures. This ensured that color variations corresponded exclusively to the specific condition tested and prevented cumulative effects.

A sample size of 10 specimens per group was used. The sample size was subsequently verified statistically as described in the Statistical Analysis section.

**Figure 1:**
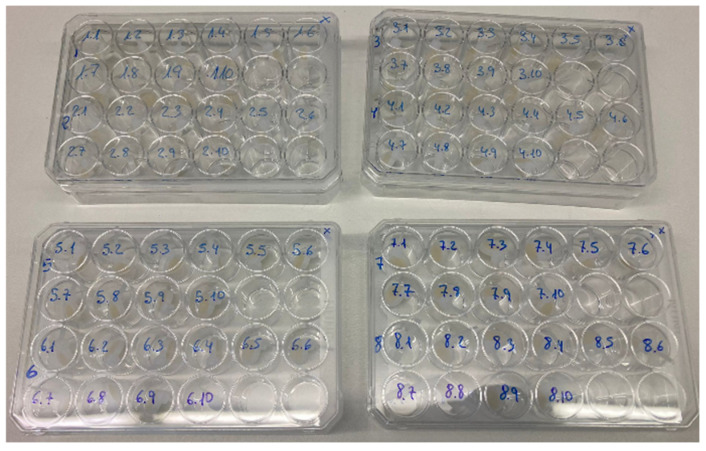
Arrangement of specimens in labeled 24-well plates according to experimental groups and immersion media.

To place the samples in contact with the different liquids, 6 × 4-well plates were used (Figure 1). Groups that shared the same procedure were placed on the same plate. Each disk was completely submerged in 2 mL of medium per well, and transparent plastic ties were used to maintain full contact between the specimen surface and the liquid. All immersion procedures were carried out at 37 °C. All specimens were left unpolished and were processed under the same standardized conditions. Surface roughness, topography, and wettability of the same CAD-CAM PMMA and graphene-reinforced PMMA materials manufactured under identical conditions have been previously reported [12,13,14,15,16,17,18].

**Figure 2:**
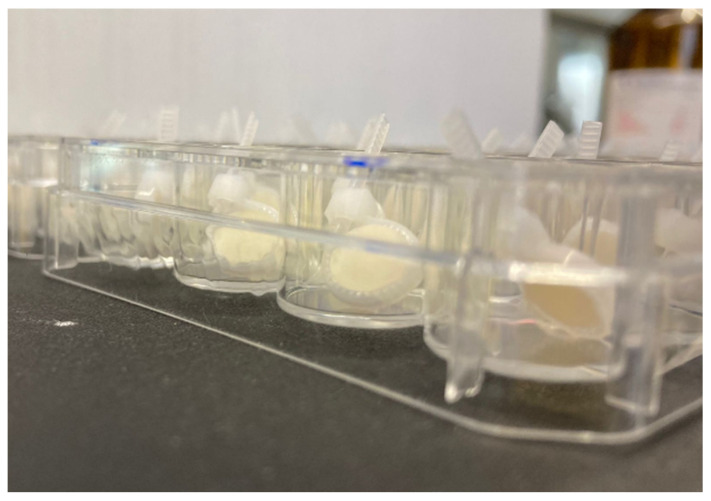
Samples placed inside the wells.

Complete immersion of the disks was performed in each testing medium (Figure 2).

### 2.2. Media Used and Method of Obtaining

The liquid media used were coffee and artificial saliva. Distilled water was used for washing the samples between media changes.

Coffee

To prepare the coffee, 2 g of soluble coffee (Nescafé^®^ Classic Natural Soluble (Nestlé España S.A., Barcelona, Spain)) was used for every 100 mL of water, previously heated in the microwave for 1 min.

The coffee solution was freshly prepared and renewed according to the experimental protocol.

Saliva

In this study, artificial saliva provided by the Gran Farmacia Galisteo Cano (Murcia, Spain) was used, produced with a magisterial formula already used in previous studies (Sorbitol 3%, Carmelosa Sodium 1%, Potassium Chloride 0.12%, Nipagin Sodium 0.1%, Sodium Chloride 0.084%, Potassium Phosphate di-Basic 0.017, Anhydrous Calcium Chloride 0.015%, Magnesium Hexahydrate Chloride 0.005, Purified Water c.s.). Three types of saliva were used depending on their pH: acidic saliva at pH 5.04, neutral saliva at pH 7.0 and basic saliva at pH 7.4.

Distilled water

Each time the media of the samples were changed, the samples were washed with distilled water and dried to remove any remnants of the previous media, to prevent the mixing of the media. For optimal washing, the liquid was removed from the wells with an extraction pump, and the samples were dried with absorbent paper. Subsequently, they were immersed in plenty of distilled water inside the wells for 2 min and dried again with paper.

### 2.3. Procedure

The experimental procedure included initial storage, exposure protocols, and daily cycling procedures.

**Figure 3:**
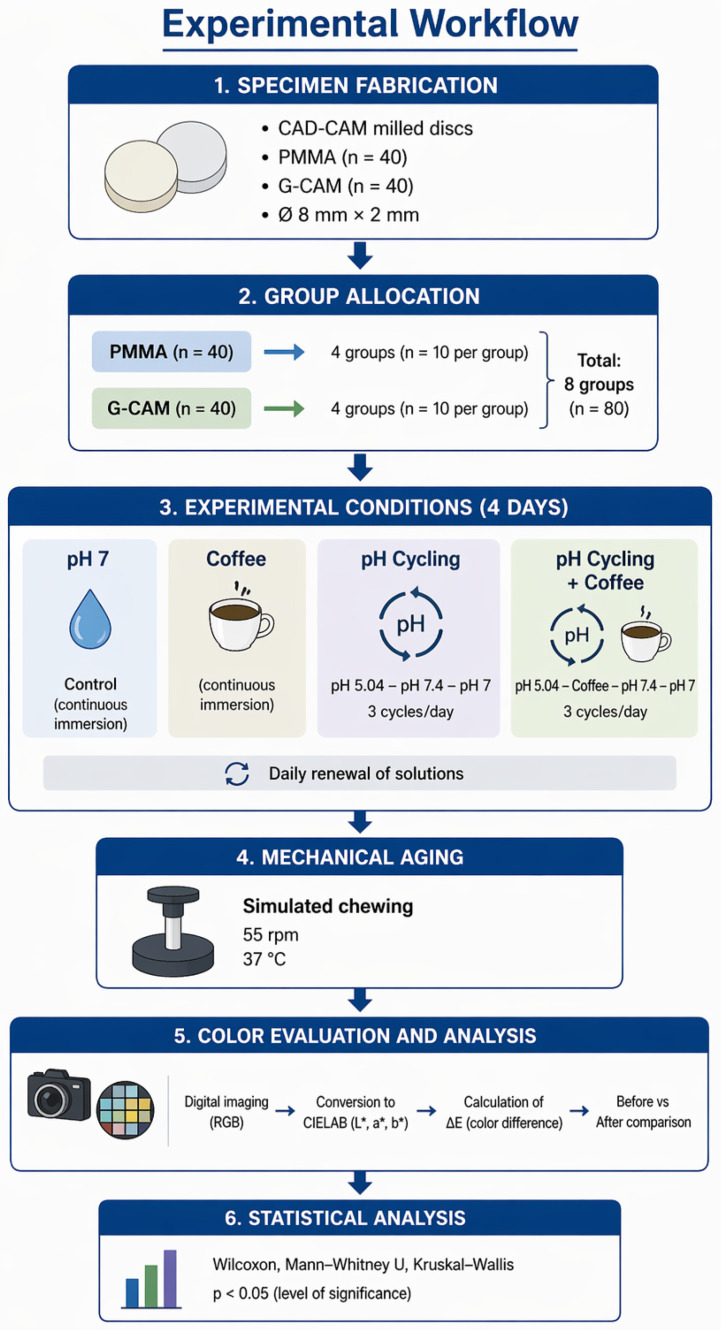
Schematic representation of the experimental workflow, including pH variation cycles, coffee immersion, washing procedures, and simulated chewing movements.

All samples were immersed in artificial saliva at pH 7 for 72 h, and they were stored in humidity at 37 °C inside the Infors Multitron agitator (INFORS HT, Bottmingen, Switzerland), but without activating the movement. When not undergoing experimental procedures, all specimens were stored in artificial saliva at pH 7 under controlled conditions (37 °C, humid environment) to simulate intraoral conditions and maintain sample stability throughout the experimental period. This machine could produce a stirring at 55 revolutions per minute to simulate the movement of chewing. Each process, described below, within each group was repeated for 4 days in a row (Figure 3).

The experimental protocol for each group is described below in a stepwise manner.

Groups 1 and 2

The samples were in contact with pH 7 saliva throughout the procedure, which was changed and renewed every 24 h.

Groups 3 and 4

The samples were in contact with coffee throughout the procedure, which was changed and renewed every 24 h.

Groups 5 and 6

In this process, pH variation was included. The specimens were agitated to simulate chewing, and the cycle was repeated three times per day to reproduce repeated daily pH fluctuations. Each cycle consisted of introducing the samples into artificial saliva at pH 5.04, stirring for 10 min, washing with distilled water for 2 min, and drying. The samples were then transferred to artificial saliva at pH 7.4, agitated for 10 min, washed with distilled water for 2 min, and dried. Finally, the samples were placed in artificial saliva at pH 7 for another 10 min. After washing and drying, the cycle was repeated. At the end of the three cycles, the samples were returned to pH 7 saliva until the next day.

Groups 7 and 8

In these last two groups, a process was carried out in the same way as the previous one, but a change pertaining to coffee was made. So, after washing and drying the samples and after immersing them in acidic saliva with pH 5.04, the samples were immersed in coffee for 10 min, washed and dried, and then their pH was adjusted to pH 7.4, and this cycle was repeated three times each day, as in the previous group.

The experimental workflow is summarized in Figure 3 to facilitate understanding of the sequence of procedures applied to each group.

Two operators were in charge of carrying out the experiment, and two different operators performed the reading using the image service to avoid bias.

### 2.4. Photo Record

**Figure 4:**
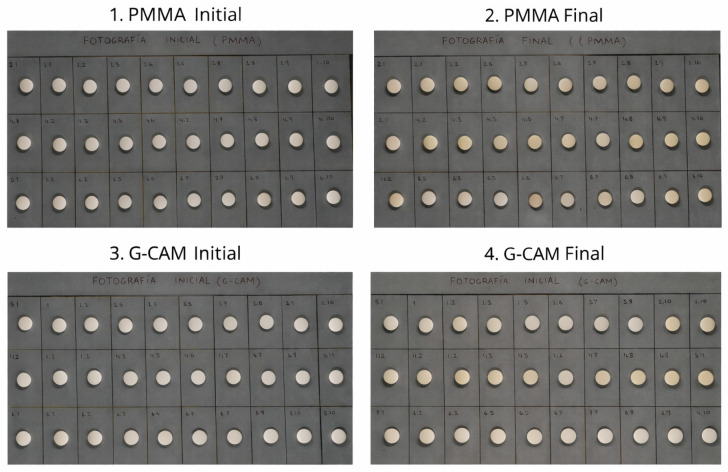
Samples after immersion.

To check for color changes, the samples were placed on a dark gray template for contrast, and photographs were taken before and after the procedures (Figure 4). A dark gray background was selected to provide consistent contrast and minimize light reflection during image acquisition. All photographs were taken under standardized conditions to ensure reproducibility of color measurements.

The photographs were taken with an image reader (Amersham™ Imager 600, GE Healthcare, Chicago, IL, USA) that has integrated software to generate and analyze the data. After taking photographs, values of grays, red, green and blue colors, hue, saturation and brightness were obtained.

The imaging system provides numerical RGB values that allow quantitative assessment of chromatic variation between initial and final measurements. These RGB data were subsequently converted into CIELAB color coordinates (L*, a*, b*) using a validated mathematical transformation based on the dataset provided by Vlad et al. (2020) Microsoft Excel for Microsoft 365 (Microsoft Corporation, Redmond, WA, USA) [19]. This approach enabled standardized color analysis in accordance with ISO TR 28642/2016 [20] recommendations. Color differences (ΔE) were calculated as the Euclidean distance between initial and final CIELAB coordinates to quantify overall chromatic variation.

### 2.5. Statistical Analysis

A descriptive analysis was performed for all quantitative variables. Data were summarized as mean and standard deviation, median, interquartile range (p25–p75), minimum, and maximum values.

Normality was assessed using the Kolmogorov–Smirnov and Shapiro–Wilk tests. Because the variables did not follow a normal distribution, non-parametric tests were used.

Initial and final values within each experimental group were compared using the Wilcoxon signed-rank test. Differences between PMMA and graphene-reinforced PMMA (G-CAM) under the same experimental conditions were analyzed using the Mann–Whitney U test. Differences among experimental conditions within each material were analyzed using the Kruskal–Wallis test.

When the Kruskal–Wallis test showed statistically significant overall differences, pairwise comparisons were performed using Mann–Whitney U tests with Bonferroni correction. Pairwise results are reported in the tables using superscript letters; values sharing at least one letter are not significantly different, whereas values with no common letters differ significantly at *p* < 0.05.

The significance level was set at *p* < 0.05. Statistical analyses were performed using Stata v14 (StataCorp LLC, College Station, TX, USA) and verified using the supplementary dataset provided as Table S1 in the Supplementary Materials. A sample size calculation for four-group comparisons using the Kruskal–Wallis test with α = 0.05 and power ≥ 80% indicated that n = 10 specimens per group provided 92.1% power.

## 3. Results

Color changes were analyzed using CIELAB coordinates and expressed as ΔE values to provide standardized and clinically interpretable assessment of chromatic variation between baseline and post-treatment measurements.

The experimental groups were analyzed according to the original predefined design, with separate reporting for each material and each experimental condition. This approach avoided pooling subgroup data and allowed condition-specific interpretation of the findings.

Table 2 presents the ΔE values for the eight original experimental groups. The overall comparison among groups was statistically significant (Kruskal–Wallis, *p* < 0.001), indicating that color change depended on both material and experimental condition.

Within PMMA, the experimental condition significantly affected color change (Kruskal–Wallis, *p* < 0.001). Coffee and pH change plus coffee produced significantly greater ΔE values than the pH 7 control. The pH change condition alone remained clinically acceptable and was not significantly different from the pH 7 control.

Within graphene-reinforced PMMA, the overall comparison among conditions was statistically significant (Kruskal–Wallis, *p* = 0.041); however, no pairwise comparison remained significant after Bonferroni correction. All G-CAM groups remained within clinically acceptable limits. Pairwise comparisons of ΔE values among experimental conditions within each material are shown in Table 3.

Table 4 shows pairwise comparisons between materials under the same experimental conditions. PMMA showed significantly higher ΔE values than G-CAM after coffee exposure and after the combined pH change plus coffee protocol. Differences between materials were not statistically significant under pH 7 or pH change alone.

Overall, the greatest color changes were observed in PMMA specimens exposed to coffee and to pH change plus coffee. These two PMMA groups exceeded the clinical acceptability threshold, whereas all G-CAM groups remained below this threshold.

These results indicate that the color stability advantage of graphene-reinforced PMMA was most evident under staining conditions involving coffee, particularly when combined with pH variation.

## 4. Discussion

Color stability is a clinically relevant parameter for provisional restorative materials, as discoloration may compromise esthetic acceptability during intraoral service. The present in vitro study compared conventional PMMA and graphene-reinforced PMMA using ΔE values derived from CIELAB coordinates, allowing interpretation of the results in relation to clinical perceptibility and acceptability thresholds [5,19,20,21,22,23,24].

The main finding was that color change depended on both material and exposure condition. Conventional PMMA showed the highest ΔE values when exposed to coffee and to the combined pH change plus coffee protocol. These groups exceeded the clinical acceptability threshold, indicating that the observed discoloration would likely be clinically relevant [15]. In contrast, graphene-reinforced PMMA remained within clinically acceptable limits under all tested conditions.

The results therefore partially support the working hypothesis. Graphene-reinforced PMMA showed lower color change than conventional PMMA in the coffee-containing protocols, particularly under the combined pH change plus coffee condition. However, differences between materials were not statistically significant under pH 7 or pH change alone, suggesting that the benefit of graphene reinforcement is more evident when the material is challenged by staining agents rather than by pH variation alone.

The greater discoloration observed in PMMA after coffee exposure may be explained by the interaction between the polymer matrix and chromogenic compounds present in coffee, such as tannins and polyphenols. These substances can adhere to or penetrate polymer surfaces, especially when surface irregularities or water sorption facilitate pigment retention. The marked ΔE increase in the PMMA coffee and pH change plus coffee groups is consistent with this mechanism [11].

The combined pH change plus coffee condition produced the greatest discoloration in conventional PMMA. This finding suggests that pH fluctuations may increase susceptibility to staining when followed by exposure to chromogenic media. Acidic conditions may promote superficial softening or changes in water sorption, thereby facilitating pigment uptake during subsequent coffee exposure. In the present study, this effect was clinically relevant for PMMA but not for graphene-reinforced PMMA [17,19,25,26,27,28].

The improved behavior of graphene-reinforced PMMA under coffee-containing conditions may be related to the modification of surface and structural properties produced by graphene incorporation. Graphene-based reinforcement has been associated with improved mechanical behavior and potentially lower water sorption or surface degradation in polymer-based materials. These characteristics may reduce pigment retention and help explain the lower ΔE values observed in G-CAM [24].

Nevertheless, the present findings should be interpreted cautiously. The study evaluated color change in standardized disk-shaped specimens under controlled in vitro conditions. Although this design allowed direct comparison between materials and exposure protocols, it does not fully reproduce the geometry, polishing procedures, oral biofilm, intermittent dietary exposure, salivary flow, or long-term aging of clinical provisional restorations.

The use of complete immersion was useful for standardizing exposure and maximizing contact between the specimens and testing media, but it represents a limitation because clinical restorations are exposed to staining agents intermittently. Similarly, the use of unpolished specimens improved standardization but does not fully reproduce clinical finishing and polishing procedures. These factors should be considered when translating the results to clinical practice [16].

Another limitation is that color analysis was performed using an imaging system with RGB-to-CIELAB conversion rather than direct spectrophotometric measurement. Although a validated mathematical transformation was applied, spectrophotometry remains the reference method for dental color evaluation. Future studies should confirm these findings using spectrophotometric assessment, longer aging periods, thermocycling, mechanical brushing, and clinically finished restorations [29].

From a clinical perspective, the present results suggest that graphene-reinforced PMMA may be preferable for long-term provisional restorations in esthetically demanding areas, especially in patients with frequent coffee consumption or high exposure to staining beverages. Conventional PMMA may remain suitable for short-term use or lower esthetic-risk situations, but clinicians should be aware that coffee exposure, particularly when combined with pH fluctuations, may produce clinically unacceptable discoloration. Therefore, in patients with frequent coffee intake, high exposure to staining beverages, or expected long-term provisionalization, clinicians should consider graphene-reinforced PMMA as a preferable option when color stability in esthetic areas is a priority [30,31].

Accordingly, material selection should not be based only on general mechanical properties or handling characteristics, but also on the expected duration of provisionalization, the esthetic demands of the case, and patient-related staining risk factors. In cases requiring prolonged provisionalization, G-CAM may offer a clinically relevant advantage in maintaining color stability.

## 5. Conclusions

Within the limitations of this in vitro study, graphene-reinforced PMMA demonstrated better color stability than conventional PMMA under coffee-containing staining conditions. Conventional PMMA exposed to coffee and to pH change plus coffee exceeded the clinical acceptability threshold, whereas graphene-reinforced PMMA remained clinically acceptable in all tested conditions. These findings suggest that graphene-reinforced PMMA may be a more suitable option for long-term provisional restorations in esthetically demanding situations or in patients with frequent exposure to staining beverages. Further studies using clinically finished restorations, longer aging protocols, and spectrophotometric assessment are required to confirm these results.

## Figures and Tables

**Table 1:** Classification of samples according to materials and experimental procedures.

Group	Material	Procedure
1	PMMA	pH 7
2	G-CAM	pH 7
3	PMMA	Coffee
4	G-CAM	Coffee
5	PMMA	pH change
6	G-CAM	pH change
7	PMMA	pH + coffee
8	G-CAM	pH + coffee

**Table 2:** ΔE values according to the original experimental groups, with pairwise comparison letters and clinical interpretation.

Group	Material	Condition	ΔE Median (p25–p75)	Pairwise Letter *	Clinical Interpretation
1	PMMA	pH 7	1.83 (1.55–2.69)	a	Acceptable
2	G-CAM	pH 7	2.81 (2.33–3.64)	a,b	Acceptable
3	PMMA	Coffee	4.91 (4.54–4.98)	b	Unacceptable
4	G-CAM	Coffee	2.32 (2.22–2.77)	a,b	Acceptable
5	PMMA	pH change	2.77 (2.53–3.54)	a,b	Acceptable
6	G-CAM	pH change	2.25 (2.14–2.95)	a,b	Acceptable
7	PMMA	pH + coffee	5.71 (4.40–6.05)	b	Unacceptable
8	G-CAM	pH + coffee	1.30 (1.07–2.04)	a	Acceptable

* Pairwise letters refer to the Bonferroni-adjusted comparisons among the eight original groups. Values sharing at least one letter are not significantly different; values with no common letters differ significantly (*p* < 0.05). ΔE values above 3.3 were considered clinically unacceptable.

**Table 3:** Pairwise comparison of ΔE values among experimental conditions within each material.

Material	Condition	ΔE Median (p25–p75)	Letter *
PMMA	pH 7	1.83 (1.55–2.69)	a
PMMA	pH change	2.77 (2.53–3.54)	a,b
PMMA	Coffee	4.91 (4.54–4.98)	c
PMMA	pH + coffee	5.71 (4.40–6.05)	b,c
G-CAM	pH 7	2.81 (2.33–3.64)	a
G-CAM	pH change	2.25 (2.14–2.95)	a
G-CAM	Coffee	2.32 (2.22–2.77)	a
G-CAM	pH + coffee	1.30 (1.07–2.04)	a

* Within each material, values sharing at least one superscript letter are not significantly different; values with no common letters differ significantly after Bonferroni correction (*p* < 0.05).

**Table 4:** Pairwise comparison of ΔE values between PMMA and graphene-reinforced PMMA under the same experimental conditions.

Condition	PMMA ΔE Median (p25–p75)	G-CAM ΔE Median (p25–p75)	*p*-Value	Interpretation
pH 7	1.83 (1.55–2.69)	2.81 (2.33–3.64)	0.089	Not significant
Coffee	4.91 (4.54–4.98)	2.32 (2.22–2.77)	0.003	G-CAM lower ΔE
pH change	2.77 (2.53–3.54)	2.25 (2.14–2.95)	0.186	Not significant
pH + coffee	5.71 (4.40–6.05)	1.30 (1.07–2.04)	0.001	G-CAM lower ΔE

Mann–Whitney U test was used for comparisons between materials within each condition.

## Data Availability

The original contributions presented in this study are included in the article and Supplementary Materials. Further inquiries can be directed to the corresponding author.

## Supplementary Materials

The following supporting information can be downloaded at: https://www.mdpi.com/article/10.3390/dj14060319/s1, Table S1: Supplementary dataset containing the raw data and statistical results used for the analysis of color changes expressed as ΔE values.
